# Predicting onset of complications from diabetes: a graph based approach

**DOI:** 10.1007/s41109-018-0106-z

**Published:** 2018-11-15

**Authors:** Pamela Bilo Thomas, Daniel H. Robertson, Nitesh V. Chawla

**Affiliations:** 1 iCeNSA, Department of Computer Science and Engineering, University of Notre Dame, 384E Nieuwland Science Hall, Notre Dame, 46656 Indiana USA; 2 Indiana Biosciences Research Institute, 1345 W. 16th Street Suite 300, Indianapolis, 46202 IN USA

**Keywords:** Disease network, Diabetes, Real-world data, Heart failure, Kidney disease, Myocardial infarction, Retinopathy, Liver disease, Disease prediction

## Abstract

Diabetes is a significant health concern with more than 30 million Americans living with diabetes. Onset of diabetes increases the risk for various complications, including kidney disease, myocardial infractions, heart failure, stroke, retinopathy, and liver disease. In this paper, we study and predict the onset of these complications using a network-based approach by identifying fast and slow progressors. That is, given a patient’s diagnosis of diabetes, we predict the likelihood of developing one or more of the possible complications, and which patients will develop complications quickly. This combination of "if a complication will be developed” with ”how fast it will be developed” can aid the physician in developing better diabetes management program for a given patient.

## Introduction

Diabetes is a significant public health concern in the United States. According to the Center for Disease Control (CDC), in 2015 it was estimated that 30.3 million people have diabetes, with 23.1 million cases diagnosed and 7.2 million undiagnosed (for Disease Control et al. 2017). 90 to 95 percent of those cases are Type 2 (for Disease Control et al. 2017), which is the group that we will focus on throughout this paper. Complications (co-morbidities) related to Type 2 Diabetes Mellitus (T2DM) are the key drivers of the health impact and cost of this chronic disease. The vast majority of diabetics will experience a complication from their disease (Nickerson and Dutta 2012). Recent data shows that there were 7.2 million hospital discharges reported for people with diabetes in 2014 (for Disease Control et al. 2017). Further, diabetes was ranked as the seventh leading cause of death in the United States in 2015, with the total direct and indirect cost of diagnosed diabetes in 2012 at 245 billion dollars (for Disease Control et al. 2017). It is critical to not only diagnose the onset of diabetes but also predict the onset of complications (co-morbidities), which would better assist in long-term care management, and better health and wellness for the patients.

To achieve the objective of predictability of onset of complications, we first represent a patient’s disease history as a network based on what happens in the second year after a diabetes diagnosis. Genetic determinants and other independent accelerating factors of the complications of diabetes (Brownlee 2005) clearly establish the basis for these co-morbid conditions developing over time. Furthermore, we label patients as either slow or fast progressors in developing complications arising from diabetes, thus developing sub-networks of disease evolution.

The proposed network developed in this study will not only provide a useful modeling construct but also a mechanism for visualizing disease complications. The use of networks to understand disease progression has been studied before, such as in Alzheimer’s (Wilkosz et al. 2010) and heart failure (Nagrecha et al. 2017). However, the novelty of our approach lies in the consideration of a heterogeneous network that includes nodes for disease diagnoses, tests, demographics, etc. Through the proposed networks-based approach, physicians will be able to leverage the combined experiences of other diabetics to determine how their patients’ disease will progress. Pinpointing the risks of complication is of utmost importance for recognizing possible interventions in treatments that have the potential to delay or stop further progression.

We use a large data set comprising of Type 2 Diabetes patients in Indiana, collected over 20 years obtained through the Regenstrief Institute. This data includes both diagnosis codes taken from the International Statistical Classification of Diseases and Related Health Problems, Ninth Revision and Tenth Revision, (ICD-9 and ICD-10, respectively) and clinical laboratory test results. Researchers have had success using ICD codes to predict future disease states (Davis et al. 2010). We create networks of shared patient experiences using the sub-networks of patients and then identify common groupings of disease that have the greatest propensity of developing diabetic complications. Using both diagnoses and lab results as the nodes and edges in our network we identify those results that are most predictive of diabetic complications, thereby creating a multi-plex or heterogeneous network (Kivelä et al. 2014). This analysis allows us to answer the question: which patients are most at risk for developing what complications? We group patients into two categories — fast or slow progressors, based on whether they develop complications more quickly or more slowly than 25 percent of the population, respectively. By categorizing patients into these categories, a more efficient intervention mechanism can be developed. It also allows us to study, as future work, why certain patients are fast or slow progressors, leading to personalized interventions and treatments and improved patient outcomes.

## Methods

Predicting diabetic complications is incredibly challenging due to the inequality of healthcare consumption and the speed at which patients receive diagnoses. In our work, we posit that by establishing appropriate thresholds and choosing balanced populations, we can ensure that even patients who infrequently visit their physician can still benefit from our models.

### Data description

The Regenstrief Institute created one of the earliest electronic medical record systems in 1972 to support research and continues to handle the research use of the INPC (Indiana Network for Patient Care) database (JM Overhage and McDonald 1995). With the creation of the Indiana Health Information Exchange (IHIE) in 2004 to handle the exchange of data between Indiana’s major healthcare provider systems, the availability of data for Indiana patients within INPC has greatly increased providing a key resource to drive research using "real world environment" observations and data.

**Fig. 1:**
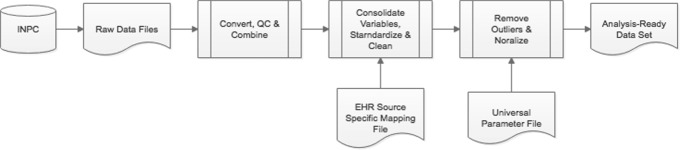
Above is a flowchart depicting the cleaning and standardization process which (1) combines and QCs the raw data files, (2) combines variables, standardizes, and cleans using a dictionary specific to the data source, and finally (3) removes variable outliers and normalizes using a universal clinical parameter dictionary

In a collaboration of Indiana Biosciences Research Institute, Regenstrief Institute, and industrial partners, a primary data set of type 2 diabetes mellitus (T2DM) patients was created. Using inclusion criteria of one T2D diagnosis code OR a laboratory glycated hemoglobin (HbA1C) test results ≥ 6.5% OR at least one Medi-Span-defined anti-diabetes medication where the patients were ≥ 18 years of age on date of first inclusion criteria. Using this criteria, a primary T2DM cohort of 805,867 individuals was identified from INPC over 20 years (1995-2015). The demographics, diagnosis codes, medical procedures, prescriptions, and results from clinical laboratory tests were extracted for these individuals(Schleyer 2016). This extracted data resulted in over 500 million records that was available for analysis. This T2DM data set was then extensively cleaned and normalized to prepare for the analyses as per the diagram in Fig. 1.

To clean this T2DM data set, the extracted INPC data placed on a secure Amazon Web Services (AWS) server. This large T2DM dataset across 20 years was multi-modal and there were many missing parameters across the records, as well as inconsistency in the measurements identified by error codes or per-patient longitudinal analysis or out of range values. In addition, we had to take into account the correction of features that were reported for quality control (QC) checks. To that end, we implemented a comprehensive a data cleaning framework to normalize the features, remove bad or missing values, and have consistent units of measure was done using PySpark. The feature values were normalized and extreme values were identified and filtered on minimum and maximum values ever measured for a parameter. Additionally, if any values were +/- 2 standard deviations from the median, they were filtered. Also, we looked for more than two distribution patterns in the data where potentially two different units of measure were applied to the same variable, which could indicate a problem with poor previous data integration. After this extensive effort to clean all the issues from this "real-world" captured data set from INPC, an "analysis-ready" data set was created for the modeling. An overview of the size of the different data tables is given in Table 1.

**Table 1:** Size and amount of data per file used

Type of data (Study cohort)	Rows	Data columns	Size
Patients	805,867	13	149 MB
ICD diagnosis codes	96,295,549	3	2.2 GB
Clinical laboratory results	388,524,849	7	393 GB

We use the following to categorize primary T2DM diagnoses and complications:

- Type 2 diabetes mellitus - ICD9/ICD10 codes 249, 250, 357.2, 362.[01-07], 366.41, E10, E11
- Kidney disease - ICD9/ICD10 codes 584, 586, 585, 403, 404, 581, 583, 588, N18, N17, N19, I12, I13, N04, N05, N08, N25, 593
- Liver disease - ICD9/ICD10 codes 571, 572, 573, K76, K75
- Heart failure - defined as ICD9/ICD10 codes 428, I50
- Myocardial infarction - ICD9/ICD10 codes 410, 412, I21
- Stroke - ICD9/ICD10 codes 435, G45, 430, 431, I60, I61, 432, I62, 436, 433, 434
- Retinopathy - ICD9/ICD10 codes 362, H35

We further sample to create the following data about patients: patient diagnosis, which contains all the diagnoses codes (ICD-9/ICD-10) received by a patient, demographics, which contains age, gender, and race/ethnicity information, and clinical variables, which contains metabolic measurements taken while at the doctor’s office. Header files for the diagnosis table is given in Table 2, patient data is given in Table 3, and clinical variables is given in Table 4. The number of patients who were diagnosed with each complication is given in Table 5.

**Table 2:** Diagnosis file - this file contains information regarding ICD codes that went along with a diagnosis received on that day

STUDYID	DX_INDEX	DX_CODE
1250869	0	250.0
1250869	411	244.9
1250869	487	I50.22
1250869	732	I50.22
1252696	0	E10.9
1252696	172	K75.81
1252696	180	K75.81
1252696	195	K75.81
1252696	209	K75.81
1252696	212	I50.9
1255288	0	250.0
1255288	43	K40.90
1255288	325	H35.30

Diagnoses can appear in subsequent visits. Day 0 is the day that a type 2 diabetes diagnosis was received

**Table 3:** The patient database contains patient age, gender, and race

STUDYID	INDEX_YEAR	INDEX_AGE	GENDER	RACE
1250869	2014	75	M	UNKNOWN/NOT DOCUMENTED
1252696	2015	53	F	UNKNOWN/NOT DOCUMENTED
1255288	2015	44	M	UNKNOWN/NOT DOCUMENTED

**Table 4:** The clinical variables file contains measurements regarding blood and urine samples during a patient visit, along with the patient age during the visit, the age at which they were diagnosed with diabetes, and the number of days after the first diabetes diagnosis the visit occurred

STUDYID	AGE	DAYS_VIS_INDEX	INDEX_AGE	albumin	alp	alt	ast	bun	chloride	…
1250869	75	0	75	4	80	93	64	18	105	…
1250869	76	411	75					17	106	…
1250869	76	487	75					26	105	…
1250869	76	732	75		79	7	14	19	108	…
1252696	52	0	53					14	107	…
1252696	52	172	53						…	
1252696	52	180	53		89	16	28	20	109	…
1252696	52	195	53					13	110	…
1252696	52	209	53							…
1100737	50	212	53					15	103	…
1255288	44	0	44	3.5	177	28	17	19	100	…
1255288	44	43	44	3.6	138	21	13	22	104	…
1255288	48	325	47					17	103	…

A full list of the columns contained is located at the end of this document under Additional file ??. Abbreviations: ALP (alkaline phosphate), ALT (alanine transaminase), AST (aspartate transaminase), BUN (blood urea nitrogen)

**Table 5:** This table provides the number of patients diagnosed with each complication included in the dataset. These patients were randomly divided into 80 percent training and 20 percent testing sets

Condition	Number diagnosed
Kidney disease	49,720
Heart failure	32,798
Stroke	30,474
Liver disease	20,761
Retinopathy	20,627
Myocardial infarction	19,930


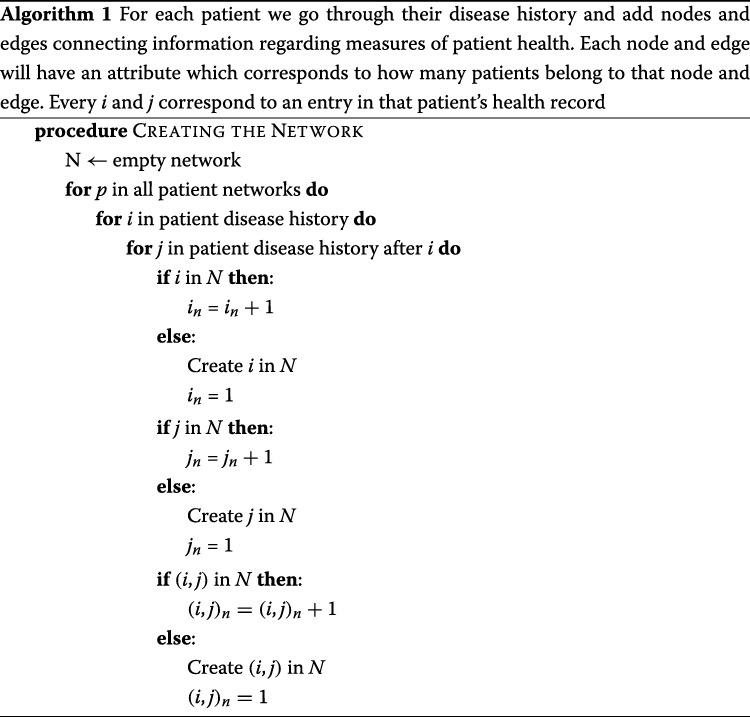


### Building disease diagnoses graphs

We detail the network construction in Algorithm 1, and network pruning in Algorithm 2. We retain a listing of the edges and nodes that represent the fast paths to diabetic complications, along with the nodes that result in the largest information gain.


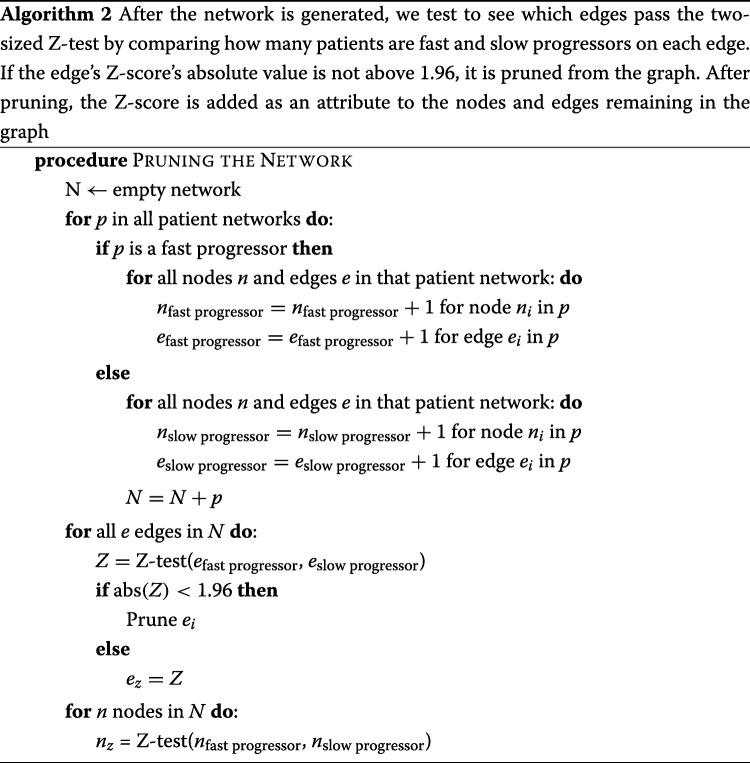


There are three primary data sources that we use to build our models: patient demographic data, which remains constant throughout the duration of the study and is represented by nodes at the beginning of the network at time zero; patient diagnosis, which contains all the diagnoses that occur over the course of a patient’s visit with a doctor or healthcare provider; and clinical variables, which contain all the available measurements and laboratories tests available in the patient’s health records as contained in INPC.

We tested the following clinical variables and grouped them into quartiles, which were included in the clinical variables file: non high-density lipoprotein cholesterol (Non-HDL C), low-density lipoprotien (LDL) high-density lipoprotein (HDL) ratio, thyroid-stimulating hormone (TSH), fibrosis-4 (Fib 4) index, total cholesterol, low-density lipoprotein cholesterol (LDL C), high-density lipoprotein cholesterol (HDL C), cholesterol ratio, total bilirubin, basophil platlet count (PC), monocyte count, aspartate transaminase to platelet ratio index (APRI), neutrophil count, albumin, alkaline phosphatase (ALP), aspartate transaminase (AST) alanine transaminase (ALT) ratio. eosinophil PC, protein, HbA1C, ALT, estimated glomerular filtration rate (eGFR), AST, lymphocyte PC, calcium, red blood PC, platelet count, mean corpuscular volume (MCV), mean corpuscular hemoglobin (MCH), glucose, blood urea nitrogen (BUN), chloride, creatinine, and carbon dioxide (CO2).

**Fig. 2:**
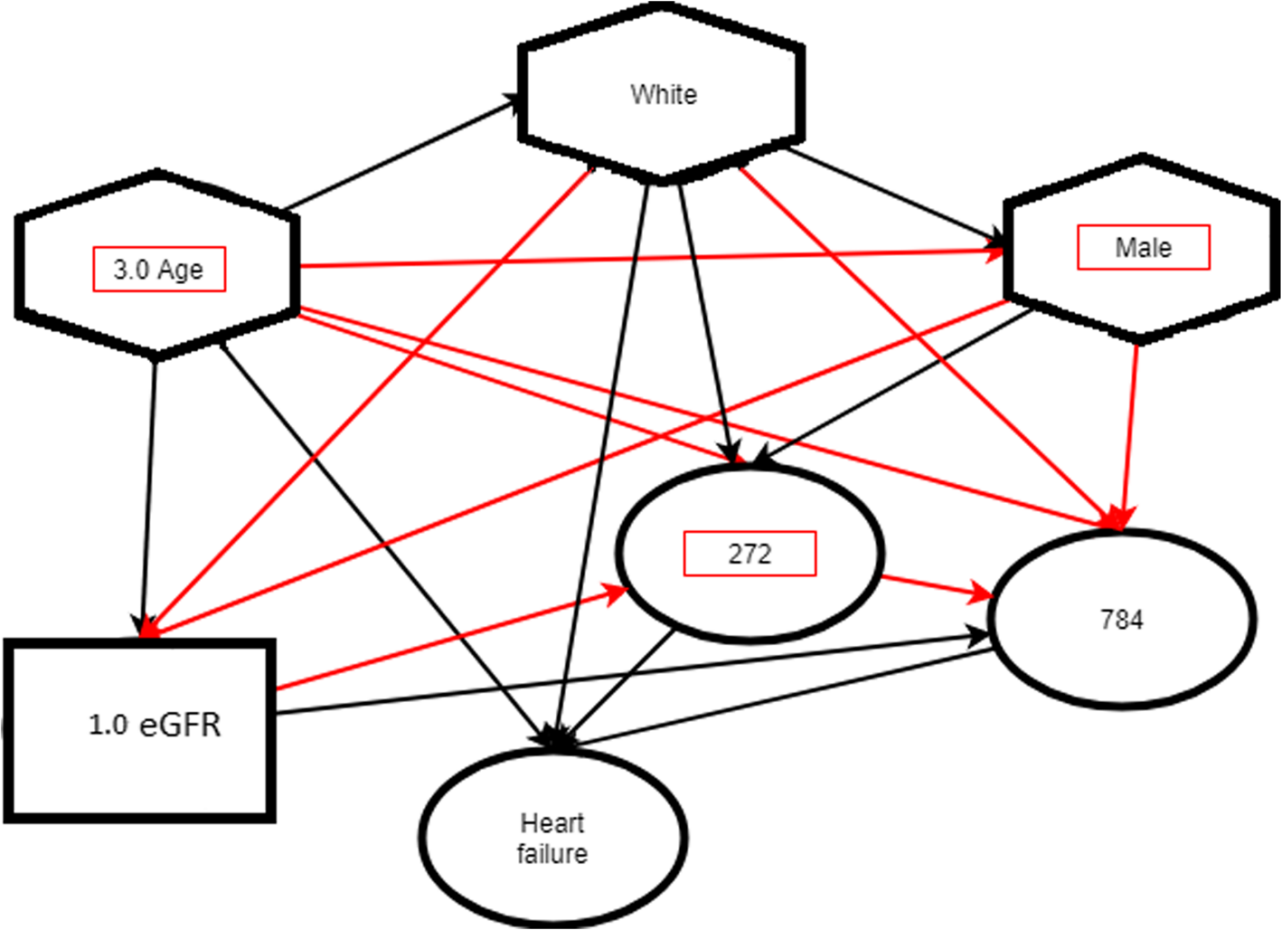
Above is an example of a patient network which contains demographic information, lab results, and diagnoses codes, for a patient who develops heart failure as a fast progressor. The most significant edges and nodes, as determined by the two-sided Z-test, marked in red, are used in patient risk calculation. Circles represent ICD diagnoses, hexagons demographic information, and squares clinical variables. Age and clinical variables had been quartiled such that 3.0 Age represents a patient whose age is in the top 75 percent of patients, and where 1.0 eGFR represents someone whose eGFR is between 25-50 percent when compared to the patient population

Additionally included in the clinical variables file were the following variables, pre-processed into normal and abnormal statuses: weight classification, HDL C, high serum creatinine, high urine glucose, hyperglycemia, hypertension, hypertriglyceridemia, impaired fasting glycemia (IFG), impaired glucose tolerance (IGT), LDL C, and triglycerides. Finally, we also quartile the age of the patients so that we have large groups to test on. Then every piece of information in a patient history is linked all other nodes, thus creating a heterogeneous network. An example of the network is given in Fig. 2.

After building the network, we prune it by discarding any edges that do not contain statistically significant differences between the fast and slow progressors as defined by using a two-proportion Z test score.

**Fig. 3:**
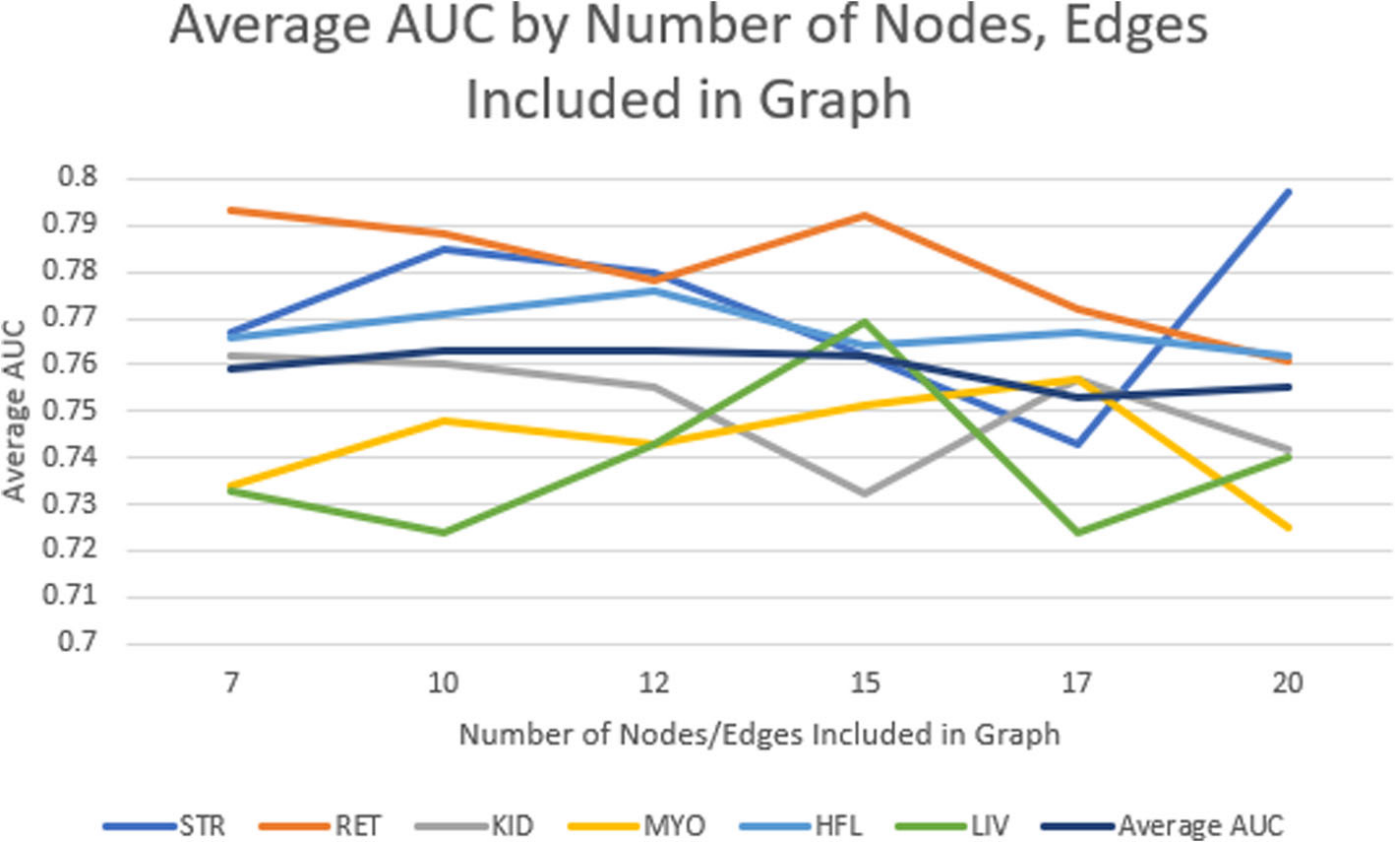
Above is a graph of the values shown in Table 6

To determine if a patient is a slow or fast progressor, the nodes and edges of the sub-network that match the patient’s medical history are traversed and their individual probability of developing a complication is computed. We assume that the node and edge weights, corresponding to the percentages of patients who suffer from that complication that are contained by that node or edge, are equally likely and statistically independent. These weights are multiplied together to get the probability of being a fast progressor. To decrease noise, we experimentally concluded that the weights, or percent likelihood of developing the specific complication of diabetes, corresponding to the top 12 most significant edges and nodes are used as determined by the two-proportion Z-test. In other words, for each individual patient, we only used the most significant parts of their individual network to predict whether or not that patient was a fast or slow progressor. The average AUC values from each of these experiments is shown in Table 6 and Fig. 3. The weight that corresponds to the lowest probability of developing complications is removed since it was observed that removing this weight boosts the signal of the nodes and edges that result in fast progression of disease. The pruning process can be shown by referring to Fig. 2.

**Table 6:** Average area under the curve (<*AUC*>) values and Standard Deviations (<*STD*>) for each complication of diabetes based upon the number of significant nodes and edges that were included in the prediction

Nodes/Edges	STR	RET	KID	MYO	HFL	LIV	<*AUC*>	<*STD*>
7	0.767	0.793	0.762	0.734	0.766	0.733	0.759	0.021
10	0.785	0.788	0.760	0.748	0.771	0.724	0.763	0.022
12	0.780	0.778	0.755	0.743	0.776	0.743	0.763	0.016
15	0.762	0.792	0.732	0.751	0.764	0.769	0.762	0.018
17	0.743	0.772	0.757	0.757	0.767	0.724	0.753	0.016
20	0.797	0.761	0.742	0.725	0.762	0.740	0.755	0.023

We see that using the 12 most significant nodes and edges gives us the highest average AUC with the lowest standard deviation across the various complications. Abbreviations: Myocardial infarction (MYO), Heart failure (HFL), Kidney disease (KID), Liver disease (LIV), Retinopathy (RET), Stroke (STR)

The method to compute the probability that an individual will be a fast or slow progressor is: Let *w*_0_,...,*w*_*n*_ correspond to the *n* most significant edge and node weights as determined by the two-proportion Z-test, where *n*≤12. Remove *w*_*h*_ from the computation, which corresponds to the lowest probability of developing the complication. Let $p_{t} = \Pi _{i = 0}^{n}w_{i}$, and $p_{f} = \Pi _{i = 0}^{n}(1-w_{i})$. Then, the probability that a particular patient is a fast or slow progressor is $\frac {p_{t}}{p_{t} + p_{f}}$

### Data cleaning

**Fig. 4:**
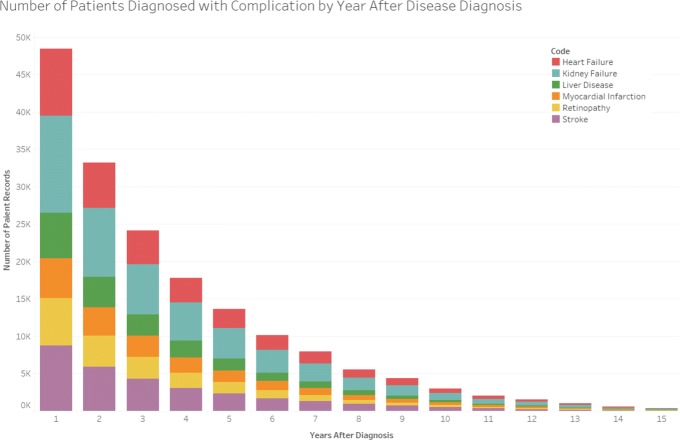
By graphing how many patients are diagnosed with each complication rate per year (starting one year after a diabetes diagnosis), we can see that most patients develop complications quickly. We want to identify what will delay complication onset by comparing the patients who are slow and fast progressors, with the fast progressors occupying the left hand side of the chart. Abbreviations: Myocardial infarction (MYO), Heart failure (HFL), Kidney disease (KID), Liver disease (LIV), Retinopathy (RET), Stroke (STR)

Only information in patient history that occurred in the second year following a Type 2 diabetes diagnosis is considered. Healthy patients survive longer than sickly ones, so if we extend our analysis for too long after a diabetes diagnosis, the data will become biased towards healthy patients. Patients tend to move and change doctors, and analyzing what occurs in the second year after the diagnosis will ensure that many patients are still in the system. We can see in Fig. 4 that many complications of diabetes occur early, so it is acceptable to limit our analysis to the that year. Our “fast progressors” all develop complications within two years of a diabetes diagnosis. Only the second year is important to us. We do not consider what occurs in the first year after diagnosis because we want to introduce more stability into our data, to exclude patients who might be in an emergency-room type situation when diagnosed.

We only consider new diagnoses that occur after a diabetes diagnosis. We do not consider diagnoses or lab values that occurred before the type 2 diabetes diagnosis. Incorporating past values might be included in future work.

- Diagnoses are truncated to the first three digits of the ICD-9 or ICD-10 code to remove the disease subtypes and only focus on the primary diagnoses.
- All nodes that are not shared by at least one percent of the population are removed.
- All patients that have received less than five diagnoses or more than twice the median amount of diagnoses are removed. This assists with biases introduced by individuals having an excessive medical history or too few observations.
- The cleaned dataset is sampled to ensure that our fast and slow progressors have the same number of patients.
- The significance on the edges is computed and any edges that do not test for a two-proportion z-test with 95 percent confidence are removed.
- Fast progressors are defined as patients who develop a complication of diabetes faster than 75 percent of the population. All patients from our dataset who develop the complication before being diagnosed with diabetes, or up to one year afterwards are removed.
- Slow progressors are defined as patients who develop a complication of diabetes slower than 75 percent of the population. Everyone retained in our network is eventually diagnosed with the complication which assists in making sure the datasets are balanced and with limited bias.
- Every node and every edge is given a Z-score, which corresponds to the likelihood of a significant difference between fast and slow progressors. Every node and edge will be given the percent likelihood that a patient who has the condition given in the node, or combination of conditions as represented by an edge, will be a fast or slow progressor.

## Results

Our test set contained 20 percent of our patients. The percent likelihood of their complication development was computed against the patient network generated from the 80 percent training set. We queried the large network for nodes and edges corresponding to an individual patient’s disease history. Because all the edges that failed to show a significant difference between the fast and slow progressors were pruned, the sub-network might be disconnected. The top five conditions that lead to each complication by percentage of fast progressors and Z-score are given in Table 7.

**Table 7:** Here we have some of the health conditions that are most likely to lead to complications based upon percentages of patients with that condition that are fast progressors, and Z-scores which correspond to the Z-test result on these particular nodes between fast and slow progressors

Health conditions likely to lead to diabetic complications			
Kidney disease	Fast %	Kidney disease	Z-score
0.0 eGFR	92	3.0 Age	18.21
Other organic psychotic conditions (chronic)	85	1.0 eGFR	16.86
Organic sleep disorders	84	0.0 eGFR	16.24
Pain not elsewhere classified	83	Heart failure	15.14
1.0 eGFR	83	Organic sleep disorders	16.01
Myocardial Infarction	Fast %	Myocardial Infarction	Z-score
Pain not elsewhere classified	82	Chronic renal failure	12.46
Chronic renal failure	82	Other forms of chronic ischemic heart disease	11.56
Other organic psychotic conditions (chronic)	81	Heart failure	11.44
0.0 eGFR	81	Hypertensive renal disease	9.96
Hypertensive renal disease	81	3.0 Age	9.92
Heart failure	Fast %	Heart failure	Z-score
Chronic pulmonary heart disease	85	3.0 Age	18.68
Chronic renal failure	85	Chronic renal failure	16.38
Other organic psychotic conditions (chronic)	85	Cardiac dysrhythmias	14.88
Other cerebral degenerations	85	Symptoms involving respiratory system and other chest symptoms	13.97
0.0 eGFR	84	Symptoms involving skin and other integumentary tissue	12.31
Liver disease	Fast %	Liver disease	Z-score
Other cerebral degenerations	88	Organic sleep disorders	11.38
Pain not elsewhere classified	87	Pain not elsewhere classified	9.81
Organic sleep disorders	87	Nonspecific abnormal findings on radiological and other examination of body structure	9.23
Acute renal failure	79	2.0 eGFR	8.48
Chronic renal failure	78	Other diseases of lung	8.41
Retinopathy	Fast %	Retinopathy	Z-score
Neoplasm of uncertain behavior of other and unspecified sites and tissues	82	3.0 Age	12.39
Renal failure unspecified	82	Disorders of lipid metabolism	10.58
Pain not elsewhere classified	81	Symptoms involving respiratory system and other chest symptoms	9.61
Other organic psychotic conditions (chronic)	81	Other and unspecified disorder of joint	9.15
Chronic renal failure	80	Heart failure	8.68
Stroke	Fast %	Stroke	Z-score
Other organic psychotic conditions (chronic)	87	3.0 Age	16.67
Other cerebral degenerations	86	Chronic renal failure	13.42
Pain not elsewhere classified	86	Symptoms involving respiratory system and other chest symptoms	12.09
Senile and presenile organic psychotic conditions	85	Symptoms involving nervous and musculoskeletal systems	11.75
Chronic renal failure	84	Symptoms involving skin and other integumentary tissue	11.56

The results for these predictions of fast progressors for onset of these various diabetic complications are shown in Table 8. These values are averaged over five runs of different test/train splits and they are comparable to the AUCs of other real-world predictive models (Weng et al. 2017).

**Table 8:** Average AUC value, specificity, and sensitivity after five experiments

Complication	AUC	Sensitivity	Specificity
Myocardial infarction	0.743	0.799	0.483
Heart failure	0.776	0.851	0.481
Kidney disease	0.755	0.774	0.558
Liver disease	0.743	0.710	0.611
Retinopathy	0.778	0.897	0.432
Stroke	0.780	0.873	0.447

## Discussion

Diabetic complications are often correlated with one another, which might reflect the generalized damage that the body has taken from a micro and macrovascular perspective (Forbes and Cooper 2013). Others have found evidence of biomarkers that have an impact on diabetic progression and can lead to a greater understanding of a patient’s personalized developments with diabetes (Scirica 2017). Other researchers have created models of diabetic risk from searching endocrinology text books and literature from clinical trials to search for indicators that lead to complications (Sangi et al. 2015). We believe that our model is unique in its ability to distinguish between fast and slow progressors.

### Similarities in Comorbid complications

Many of the top confidence nodes are shared between different complications. Correlations between the fast and slow progressors are given in Table 9. Some of the most significant nodes, including mental diseases such as psychoses, cerebral degenerations, psychotic conditions, and pain, are symptoms or causes of uncontrolled diabetes. This could be because many diabetic patients are suffering from many of the same co-morbidities which have a negative influence on disease control and care (Magnan et al. 2015). Others have found patterns of these co-morbidities, and split diabetics into several classes which represent their progression through diabetes: severe cardiac, cardiac, noncardiac vascular, risk factors, and no concordant co-morbidities (Magnan et al. 2018). Being diagnosed with a mental disorder soon after a diagnosis with diabetes would have a limiting effect on the patient’s ability to maintain glycemic control. Chronic pain also limits the control of patients’ diabetes, potentially resulting in development of complications (Krein et al. 2005). Diseases such as arthritis can impair patient function and drive barriers to lifestyle changes and regimen adherence (Piette and Kerr 2006). Other disabling conditions, such as heart failure or dementia, make self-care impossible (Piette and Kerr 2006). Lack of sleep worsens glucose tolerance (DJ et al. 2005), which could lead to fast complication development. Also, diabetic patients are at higher risk for sleep disorders such as nocturia, neuropathic pain, and restless leg syndrome (DJ et al. 2005). Patients with further developed complications could be more likely to have these problems, which lead to sleep disorders. For many patients, diabetic complications do not occur unexpectedly. It is a pattern of poor health that leads to many co-occurring complications of diabetes. Low eGFR is shown to be one of the top confidence nodes for fast progressors in kidney disease, in both the highest distinguishers and absolute percentages. Low eGFR is one of the most important markers of kidney disease (Levey and Coresh 2012). Renal function is a prognosticator of heart failure since it is a good marker for impaired hemodynamic status and general vascular disease (Hillege et al. 2006).

**Table 9:** Correlations between fast progressors of each complication

	Myocard Inf	Heart Fail	Kidney	Liver	Retinopathy	Stroke
Myocard Inf		0.688	0.576	0.367	0.312	0.528
Heart failure	0.677		0.545	0.419	0.281	0.437
Kidney	0.576	0.545		0.575	0.307	0.438
Liver	0.367	0.419	0.575		0.267	0.350
Retinopathy	0.312	0.281	0.307	0.267		0.291
Stroke	0.528	0.437	0.438	0.350	0.291	

### Potential implications for personalized medicine

In our future work, we would like to examine the false positives and identify what causes them to not develop complications immediately, even though their diagnosis history and lab results identify them as fast progressors. This will inform health management strategies – lifestyle, behavioral or environmental factors – in addition to the medication to manage diabetes. We believe this analysis should help enable recommendations for diabetic patients to limit development of complications.

## Conclusion

Given a patient’s disease history and lab results, we can predict their likelihood of developing complications from diabetes. We also show what disease diagnoses or lab results (from our heterogeneous network or graph) are most likely to lead to specific diabetic complications. We reaffirm that diabetes is a complicated disease. It continues to be important for diabetic patients to manage their disease and be aware of the complications. The diagnoses graphs can help illuminate health problems faced by many patients and what might be the best course of disease management. Not managing complications, especially for fast progressors, can cause rapid development of uncontrolled diabetes, from which it is hard to recover. Moreover, disease diagnoses graphs can also be a useful tool for physicians to understand the effects of co-morbid conditions, and personalize a wellness and disease management plan. This can lead to an improvement in both individual and population health outcomes.

## Appendix

### Data Columns Included in the Clinical Variables File

Below is a list of data columns included in the clinical variables file: STUDYID, AGE, DAYS_VIS_INDEX, GENDER, INDEX_AGE, angiotensin converting enzyme (ace), acetaminophen, acetone, act, albumin, albumin_creatinine_ratio, albumin_globulin_ratio, alcohol_pc, aldolase, aldosterone, alp, alp_bone_isoenzyme, alpha_1_antitrypsin, alpha_1_globulin, alpha_2_globulin, alpha_tocopherol, alt, ammonia, amylase, anion_gap, aorta_sinuses_diam, aortic_root_diam, aov_peak_pressure, aov_peak_velocity, apri, arterial_diastolic_bp, ast, ast_alt_ratio, antithrombin iii (atiii), band count (cnt), band_pc, bard_score, base_excess, basophil_count, basophil_pc, beta2_microglobulin, beta_globulin, beta_hydroxybutyrate, bicarbonate, blast_count, blast_pc, body mass index (bmi), body_surface_area, bun, bun_cr, bun_post_dialysis, bun_pre_dialysis, complement 3 (c3), complement 4 (c4), c_peptide, calciferol, calcium, calcium_albumin, carboxyhemoglobin, cyclic citrullinated peptide (ccp), cluster of differentiation (cd) 2_t_cells, cd3_t_cells, cd4_cd8_ratio, cd4_helper_t, cd4_t_cells, cd8_supprs_t_cells, cd8_t_cells, carcinoembryonic antigen (cea), cell_count, chloride, cholecalciferol, cholesterol_ratio, creatine kinase (ck)_bb), ck_index, ck_mb, ck_mb_tot, ck_mm, ck_total, chronic kidney disease (ckd)_stage, co2, colony_count, conjugated_bilirubin, cortisol, creatinine, creatinine_ck, creatinine_clear, c-reactive protein (crp), central venous pressure (cvp), d_dimer, (dehydroepiandrosterone) dhea_s, diabetic_nephropathy_status, diabetic_status, diastolic_bp, diastolic_bp_standing, direct_bilirubin, epstein-barr (ebv)_antibody, eGFR, eosinophil_count, eosinophil_pc, esr, estradiol_unconjugated, estrogen, factor_viii_activity, fasting_glucose, forced expiratory flow (fef)25_75, ferritin, fib_4_index, fibrinogen, fraction of inspired oxygen (fio2), folate, free_lambda, fructosamine, follicle-stimulationg hormone (fsh), gamma-glutamyl transpeptidase (ggt), globulin, glucose, glucose_gtt_1h, glucose_gtt_1hr_ob, glucose_gtt_2h, glucose_gtt_3h, glucose_gtt_pp, hba1c, hdl_c, hdl_c_status, hdl_ cholesterol (chol), hdl_ldl, height, hepatitis (hepb)_ab, hemoglobin (hgb), hemoglobin a2 (hgb_a2), high_serum_creatinine_status, high_urine_glucose_status, histamine, homeostatic model assessment of beta cell function (homa_b), homeostatic model assessment of insulin resistance (homa_ir), homocysteine, hyperglycemia_status, hypertension_status, hypertriglyceridemia_status, ifg_status, immunoglobulin a (iga), immunoglobulin e (ige), insulin-like growth factor 1 (igf_1), immunoglobulin g (igg), immunoglobulin m (igm), igt_status, immature_granulocytes_pc, indirect_bilirubin, insulin, iron, interventricular septum (ivs)_thickness, left atrium (la)_diameter, lactate, lactate_dehydrogenase, lactic acid dehydrogenase (ldh)_1, ldh_2, ldh_3, ldh_4, ldh_5, ldl_c, ldl_c_status, ldl_hdl_ratio, lh, lipase, lipoprotein (lpa), left ventricle (lv)_mass, lv_stroke_volume, lv_systolic_volume, left ventricular outflow tract (lvot)_peak_gradient, lvot_peak_velocity, left ventricular posterior wall (lvpw)_thickness_diastolic, lymphocyte_atypical, lymphocyte_count, lymphocyte_pc, lymphocyte_reactive, lymphocyte_variant, lymphotycte cerebrospinal fluid (csf), macrophage_pc, map, mch, mcv, mean_arterial_pressure, mean_glucose_bld_ghb_test, mesothelial_cells_pc, metamyelocytes_count, metamyelocytes_pc, methemoglobin, methemoglobin_pc, mixed_mono_count, mixed_mono_pc, monocyte_count, monocyte_csf_pc, monocyte_pc, myelocyte_count, myelocyte_pc, nafld_fibrosis_score, neutrophil_count, neutrophil_pc, non_hdl_c, nucleated red blood cells (nrbc)_count, nrbc_pc, nrbc_white blood cell (wbc), N-terminal pro b-type natriuretic peptide (nt_probnp), nucleated_cell_count, oxygen (o2), oxyhemoglobin_pc, p_wave_offset, p_wave_onset, partial pressure of carbon dioxide (pco2), ph, phosphorus, platelet_count, partial pressure of oxygen (po2), poly_count, poly_pc, potassium, pr_interval, pre_diabetic_status, progesterone_17_OH, promyelocytes_count, prostate_free, prostrate_total, protein, pulse, qt_corrected, quantitative insulin-sensitivity check index (quicki), red blood cell distribution width (rdw), red_blood_cell_count_csf, red_blood_pc, renal_exocrine pancreatic insufficiency (epi)_cells, respiratory_rate, selenium, serum_osmolality, smudge_cell_count, sodium, systolic_bp, systolic_bp_standing, triiodothyronine (t3)_free, t3_total, thyroxine (t4)_free, t4_total, t_wave_axis, t_wave_offset, temperature, testosterone_free, testosterone_total, total iron binding capacity (tibc), total_bilirubin, total_cholesterol, triglyceride_hdl_ratio, triglycerides, triglycerides_status, troponin, troponin_2h, tsh, urine albumin-to-creatinine ratio (uacr), unconjugated_billirubin, uric_acid, urine_albumin, urine_ascorbate, urine_bacteria, urine_billirubin, urine_cast, urine_chloride, urine_cortisol_free, urine_creatinine, urine_creatinine_24, urine_crystals, urine_epithelial_cells, urine_gamma_globulin, urine_glucose, urine_granular_cast, urine_hgb, urine_hyaline_cast, urine_ketones, urine_microalbumin, urine_microalbumin_24, urine_microalbumin_creatinine_ratio, urine_microalbumin_creatinine_ratio_24, urine_potassium, urine_protein, urine_protein_24, urine_protein_creatinine_ratio, urine_red blood cells (rbc), urine_specific gravity (sp_grav), urine_squaous_epithelial (epi)_cells, urine_trans_epi_cells, urine_urea_nitrogen, urine_urobilinogen, urine_waxy_cast, vitamin (vit)_a, vit_b1, vit_b12, vit_d2, vit_25-hydroxyvitamin d2(d2_25_oh), very low-density lipoprotein (vldl), vldl_c, waist_circumference, wbc_count, wbc_count_csf, weight, weight_classification, zinc, CARDIOVASCULAR, NEPHROPATHY, LIVER, OUTCOME

## Abbreviations

ALP: Alkaline phosphatase
ALT: Alanine transaminase
APRI: Aspartate transaminase to platelet ratio index
AST: Aspartate transaminase
ATIII: Anithrombin III
AUC: Area under the curve
AWS: Amazon Web Services
BMI: Body Mass Index
BP: Blood pressure
BUN: Blood urea nitrogen
C3: Complement 3
C4: Complement 4
CCP: Cyclic Citrullinated Peptide
CD: Cluster of Differentiation
CDC: Center for Disease Control
CEA: Carcinoembryonic antigen
CHOL: Cholesterol
CK: Creatine kinase
CKD: Chronic kidney disease
CNT: Count
CO2: Carbon dioxide
CRP: C-reactive protein
CSF: Cerebrospinal fluid
CVP: Central venous pressure
D2_25_OH: 25-hydroxyvitamin d2
DEA: Dehydroepiandrosterone
EBV: Epstein-Barr Virus
eGFR: Estimated glomerular filtration rate
EPI: Exocrine pancreatic insufficiency
EPI: Epithelial
FEF: Forced expiratory flow
Fib 4: Fibrosis-4
FIO2: Fraction of inspired oxygen
FSH: Follicle-stimulating hormone
GGT: Gamma-glutamyl Transpeptidase
HbA1C: Glycated hemoglobin
HDL: High-density lipoprotein
HDL C: High-density lipoprotein cholesterol
HEPB: Hepatitis
HFL: Heart failure
HGB: Hemoglobin
HGB A2: Hemoglobin A2
HOMA B: Homeostatic Model Assessment of Beta Cell Function
HOMA IR: Homeostatic Model Assessment of Insulin Resistance
ICD-10: International Statistical Classification of Diseases and Related Health Problems, Tenth Revision
ICD-9: International Statistical Classification of Diseases and Related Health Problems, Ninth Revision
IFG: Impaired fasting glycemia
IGA: Immunoglobulin A
IGE: Immunoglobulin E
IGG: Immunoglobulin G
IGM: Immunoglobulin M
IGT: Impaired glucose tolerance
IHIE: Indiana Health Network Exchange
ILGF-1: Insulin-like growth factor 1
INPC: Indiana Network for Patient Care
IVS: Interventricular septum
KID: Kidney disease
LA: Left atrium
LDH: Lactinc acid dehydrogenase
LDL: Low-density lipoprotien
LDL C: Low-density lipoprotein cholesterol
LIV: Liver disease
LPA: Lipoprotein
LV: Left ventricle
LVOT: Left ventricular outflow tract
LVPW: Left ventricular posterior wall
MCH: Mean corpuscular hemoglobin
MCV: Mean corpuscular volume
MYO: Myocardial infarction
Non-HDL C: Non high-density lipoprotein cholesterol
NRBC: Nucleated red blood cells
NT-PROBNP: N-terminal pro b-type natriuretic peptide
O2: Oxygen
PC: Platlet count
PCO2: Partial pressure of carbon dioxide
PO2: Partial pressure of oxygen
QC: Quality control
QUICKI: Quantitative insulin-sensitivity check index
RBC: Red blood cells
RDW: Red blood cell distribution width
RET: Retinopathy
SP GRAV: Specific gravity
STD: Standard deviation
STR: Stroke
T2DM: Type 2 Diabetes Mellitus
T3: Triiodothyronine
T4: Thyroxine
TIBC: Total iron binding capacity
TSH: Thyroid-stimulating hormone
UACR: Urine albumin-to-creatinine ratio
VIT: Vitamin
VLDL: Very low-density lipoprotein
WBC: White blood cell
